# Severe Transient Hyperglycemia in a Prediabetic Patient during Mild Acute Pancreatitis

**DOI:** 10.1155/2015/968593

**Published:** 2015-03-30

**Authors:** Aura Diana Reghina, Silvia Craciun, Simona Fica

**Affiliations:** ^1^University of Medicine and Pharmacy Carol Davila, 050474 Bucharest, Romania; ^2^Endocrinology and Diabetes Department, Elias University Hospital, 011461 Bucharest, Romania; ^3^“Victor Babes” National Research and Development Institute of Pathology and Biomedical Sciences, 050096 Bucharest, Romania

## Abstract

A 30-year-old obese male patient had been diagnosed with diabetes mellitus due to acute hyperglycemia and ketonuria. He also presented with severe hypertriglyceridemia and high levels of serum lipase. He was initially misdiagnosed with type 1 diabetes and treated with insulin for one month. At two months from first presentation, pancreatic antibodies were negative, and the C-peptide level was normal. A1c level was 5.9% without insulin treatment. The association between diabetes mellitus and acute pancreatitis is well established. We reported a case of severe transient hyperglycemia during mild acute pancreatitis in a metabolically ill patient.

## 1. Introduction

Due to the close anatomical and functional links between the exocrine and the endocrine pancreases, any disease affecting one of these parts will inevitably affect the other [1]. The pancreas has a major role in glucose homeostasis, and pancreatitis is the most frequent disease of the pancreas. Acute pancreatitis is an inflammatory disease of the pancreas. Many causes of acute pancreatitis have been discovered, but the theories about its pathogenesis are controversial. In developed countries, obstruction of the common bile duct by stones (38%) or alcohol abuse (36%) is the most frequent cause of acute pancreatitis [2]. Diabetes secondary to acute pancreatitis can be classified as type 3c diabetes mellitus, along with diabetes secondary to other exocrine pancreatic dysfunctions (relapsing and chronic pancreatitis of any etiology, hemochromatosis, cystic fibrosis, fibrocalculous pancreatopathy, pancreatic trauma, pancreatectomy, pancreatic agenesis, and pancreatic cancer) [3]. In a meta-analysis, prediabetes and/or DM were observed in 37% of individuals after acute pancreatitis, and newly diagnosed DM developed in 15% of individuals within 12 months after pancreatitis, with an increased risk at 5 years [4]. Conversely, diabetic individuals have a 92% increased risk of development of acute pancreatitis, independent of obesity, alcohol use, gallstones, and hyperlipidemia [5, 6].

## 2. Case Presentation

A 30-year-old male patient presented to our medical unit two months after he was diagnosed with type 1 DM. He had been treated with insulin for approximately one month, with the insulin dose gradually lowered until cessation due to good glycemic control.

The patient had presented to the emergency room 2 months before (for the first time in his life) with fatigue, nausea, vomiting, and epigastric pain for 3 days in the context of an excess of fat and alcohol ingestion. He also affirmed a 2-week history of polyuria, polydipsia, visual blurring, and marked physical fatigue. The patient did not report chronic but only occasional ingestion of alcohol. The patient was not aware of any other medical problems and he was not on medication prior to this episode.

The patient had no familial history of diabetes. Physical examination revealed an overweight body type (BMI = 36 kg/m^2^, weight = 108 kg, height = 174 cm), dehydration, epigastric and central abdominal pain at palpation, BP = 165/80 mmHg, and HR = 118/min.

A cardiopulmonary X-ray did not show any pathological modifications.

An abdominal ultrasound scan revealed elevated liver parameters, dilated intrahepatic biliary ducts, and mild edema of the pancreas with increased diameter in the cephalic region of 3.5 cm. At that time, blood investigations showed leukocytosis (15,300/mm^3^; normal: 4,000–10,000/mm^3^), an elevated blood sugar level (386 mg/dL), a blood cholesterol level of 646 mg/dL (normal: 150–240 mg/dL), hypertriglyceridemia (2,658 mg/dL; normal: <150 mg/dL), and a blood lipase level of 338 mg/dL (normal: <200 U/L). Urinalysis showed glycosuria, mild proteinuria, and the presence of ketone bodies. Renal and liver function tests were normal.

The patient had been diagnosed with type 1 DM and discharged with these recommendations: medical nutrition therapy and insulin treatment with 3 doses of prandial insulin aspart and one dose of basal insulin glargine. He had also received antihypertensive treatment using an angiotensin receptor blocker and treatment with a statin, fibrate, an antiplatelet agent, and pancreatic enzymes.

The patient had diminished his insulin doses under medical supervision as blood sugar levels decreased, until he did not need any insulin treatment to achieve a normal blood sugar profile.

He presented to our unit for further investigation to conclude the diagnosis and to establish a subsequent plan for therapeutic conduct.

Physical examination at the second referral revealed an obese body type (BMI = 32.67 kg/m^2^) with abdominal adiposity. Note that, after being diagnosed with DM, the patient had changed his lifestyle and had lost weight (from 108 kg to 100 kg; BP = 140/85 mmHg, HR = 64/min). The complete blood count values were in the normal range, with slightly increased blood cholesterol (203 mg/dL) and mild hypertriglyceridemia (195 mg/dL). Urea and electrolyte tests, liver function tests, and thyroid function/immunological tests were all in normal ranges. All blood glucose values during hospitalization were optimal values. The A1c level was 5.9% (determined by the HPLC method).

The C-peptide level was in the normal range (1.01 ng/mL by chemiluminescent immunoassay; normal: 0.81–3.85 ng/mL) as were immunological tests specific for autoimmune diabetes: islet cell autoantibodies (ICAs) < 0.1 unit/mL (as detected by the indirect immunofluorescent method; normal: <0.1 U/mL), glutamic acid decarboxylase autoantibodies (GADAs) < 5 IE/mL (as detected by immunoenzymatic assay; normal: <10 IE/mL), and insulinoma-associated-2 autoantibodies (IA-2A) < 10 IE/mL (as detected by ELISA; normal: <10 IE/mL).

The patient received lifestyle and dietary counseling and was discharged with recommendations to continue to monitor his blood sugar and to lose weight. He also received a recommendation to continue treatment for his high blood pressure and dyslipidemia.

At almost three months after this evaluation, physical examination revealed weight 98 kg, BMI = 32.3 kg/m^2^, and normal blood pressure (under treatment). The ultrasound of pancreas was normal. Laboratory data (including cholesterol and triglycerides) were within normal range except A1c which was 5.8%, prediabetic state.

The patient provided informed consent to be described in this case report.

## 3. Discussion and Conclusion

We report the case of a young obese patient with transient hyperglycemia during acute pancreatitis.

Acute pancreatitis was suggested by the patient's symptoms at the time when he presented to the emergency unit (nausea, vomiting, and epigastric and central abdominal pain) in the context of previous alcohol and fatty food ingestion. Blood tests revealed high triglyceride, cholesterol, and lipase levels, and an ultrasound revealed mild pancreatic edema with increased diameter of the head of the pancreas. Initially, he was incorrectly diagnosed with type 1 DM due to his symptoms, severe hyperglycemia, and ketonuria as a young patient.

At the moment when the patient presented to our clinic, he had been free of insulin treatment for one month, with a good glycemic control (A1c = 5.9%). We tried to find out whether he was in the “honeymoon” period of type 1 DM or had transient hyperglycemia in the context of acute pancreatitis.

A type 1 DM diagnosis was refuted by specific immunity tests, a C-peptide level in the normal range, and the level of A1c. Although we do not have an A1c value from the first presentation to conclude whether the patient had chronic hyperglycemia prior to the acute pancreatitis episode, the actual A1c value did not correlate with the most recent blood sugar levels. Therefore, we believe that this value was the result of the hyperglycemia 2 months before.

The severity of pancreatitis (mild) in our patient was not correlated with the severity of hyperglycemia (with ketonuria), which is concordant with other studies [4].

We are not aware of the glycemic status of the patient before the first presentation but we believe he had prediabetes given that the patient was obese, dyslipidemic, and hypertensive. This prediabetes state worsened under systemic inflammatory response syndrome related to the acute pancreatitis. Improvement occurred with remission of systemic inflammatory response syndrome and weight loss. There is a bidirectional relationship between dysglycaemia and pancreatitis [4, 6, 7]. Acute pancreatitis could induce beta cell dysfunction and transient insulin deficiency and also could increase insulin resistance due to systemic inflammation. On the other hand, dysglycaemia (prediabetes and type 2 diabetes) increased the risk of acute pancreatitis. But in our case the more probable etiology of pancreatitis is acute alcohol abuse which aggravated hypertriglyceridemia.

Due to the value of A1c at second and third presentation of patient (5.9% and 5.8%, resp.) we believe that the diagnosis of type 2 diabetes at baseline is unlikely even though the patient lost 7.4% of his weight. The patient lost some weight from his initial presentation but this was too modest to explain the improvement in glycemic control. A substantial weight loss is needed for diabetes remission occurrence. Mingrone et al. have shown that diabetes remission had occurred in no patients in the medical-therapy group versus bariatric surgery and weight changes were not significant predictor of diabetes remission or of improvement in glycemia at 1 and 3 months [8].

In a young patient with severe hyperglycemia and ketonuria, even in the context of acute pancreatitis, it is of therapeutic and prognostic importance to check pancreatic autoantibodies. If pancreatic autoantibodies are positive, this parameter must be rechecked after the resolution of pancreatitis. Transient GADA positivity has been reported in patients with acute pancreatitis [9]. If pancreatic autoantibodies are negative, patients have secondary diabetes due to pancreatitis or type 2 diabetes complicated by acute pancreatitis.

The therapeutic approach consists of insulin treatment as initial therapy to restore the optimal level of glycemia and to overcome glucotoxicity. Insulin doses should be reduced according to glycemic levels as the pancreatitis resolves. Recommendations of lifestyle changes and also identification of risk factors for the association between diabetes and pancreatitis, including alcohol use, gallstones, obesity, dyslipidemia, and glycemic control, are of great importance to decrease the risk of pancreatitis relapse.

In conclusion, an obese, metabolically ill patient with prediabetes state is at increased risk of acute pancreatitis that could result in an important increase of insulin resistance and beta cell dysfunction associated with severe transient hyperglycemia which could improve after remission of systemic and pancreatic inflammation.
